# Urinary Metabolomics Study of Patients with Gout Using Gas Chromatography-Mass Spectrometry

**DOI:** 10.1155/2018/3461572

**Published:** 2018-10-16

**Authors:** Qianqian Li, Shuangshuang Wei, Dehong Wu, Chengping Wen, Jia Zhou

**Affiliations:** ^1^College of Basic Medical Science, Zhejiang Chinese Medical University, 548 Binwen Road, Hangzhou 310053, China; ^2^The Second Affiliated Hospital of Zhejiang Chinese Medical University, 318 Chaowang Road, Hangzhou 310005, China

## Abstract

**Objectives:**

Gout is a common type of inflammatory arthritis. The aim of this study was to detect urinary metabolic changes in gout patients which may contribute to understanding the pathological mechanism of gout and discovering potential metabolite markers.

**Methods:**

Urine samples from 35 gout patients and 29 healthy volunteers were analyzed by gas chromatography–mass spectrometry (GC–MS). Orthogonal partial least-squares discriminant analysis (OPLS-DA) was performed to screen differential metabolites between two groups, and the variable importance for projection (VIP) values and Student's t-test results were combined to define the significant metabolic changes caused by gout. Further, binary logistic regression analysis was performed to establish a model to distinguish gout patients from healthy people, and receiver operating characteristic (ROC) curve was made to evaluate the potential for diagnosis of gout.

**Result:**

A total of 30 characteristic metabolites were significantly different between gout patients and controls, mainly including amino acids, carbohydrates, organic acids, and their derivatives, associated with perturbations in purine nucleotide synthesis, amino acid metabolism, purine metabolism, lipid metabolism, carbohydrate metabolism, and tricarboxylic acid cycle. Binary logistic regression and ROC curve analysis showed the combination of urate and isoxanthopterin can effectively discriminate the gout patients from controls with the area under the curve (AUC) of 0.879.

**Conclusion:**

Thus, the urinary metabolomics study is an efficient tool for a better understanding of the metabolic changes of gout, which may support the clinical diagnosis and pathological mechanism study of gout.

## 1. Introduction

Gout, a common type of arthritis that features by deposits of monosodium urate crystals in the articular cavity or synovial fluid, is always accompanied by sudden joint inflammatory reaction [1–3]. The incidence of gout has reportedly experienced continuous growth over the last few decades around the world. The contemporary prevalence of gout in men is much higher than that in women and steadily increases before the age of 70 [4, 5]. In recent years, great advance has been made in investigating the pathogenesis of gout. However, there remains some uncertainty about the pathogeny of gout which raises the difficulties in its prevention, diagnosis, and treatment. The gold standard of gout diagnosis involves an invasive test of synovial fluid for the presence of monosodium urate crystals [6], but does not always work in clinical practice. It is desirable to develop a more accurate, rapid, and reliable diagnostic method on the diagnosis of gout.

Metabolomics analysis has been extensively applied in pathologic mechanism investigation and biomarker detection of diseases. As the change in metabolite levels reflects dynamic variations in an organism [7, 8], the underlying biochemical processes associated with disease can be revealed, thus providing insights to better understand the disease state [9]. The major analytical tools of metabolomics include gas chromatography–mass spectrometry (GC–MS) [10], liquid chromatography-mass spectrometry (LC-MS), and nuclear magnetic resonance (NMR) spectroscopy. Various samples from the human body (such as cells, blood, urine, and cerebrospinal fluid) can be studied by using these novel and rapid analysis techniques to aid the assessment of various diseases [11, 12].

In recent years, there have been numerous reports on the application of metabolomics in studying rheumatic immune diseases [13], such as ankylosing spondylitis [14], rheumatoid arthritis [15], systemic lupus erythematous [16], and gout [17]. However, investigations of gout by using GC-MS have been rarely reported. GC-MS is a suitable technique for comprehensive metabolomics studies which provides excellent separations for complex samples. In addition, commercial GC-MS mass spectral libraries are available for the identification of metabolites. Metabolic profiling based on GC-MS involves metabolites with different chemical structures, such as fatty acids, carbohydrates, steroids, and amines [18, 19].

In this study, GC–MS was applied to detect urinary metabolic changes in gout patients. Based on multivariate statistical analysis, the metabolic profiles of the urine from patients with gout were studied, in which characteristic metabolites were selected to provide a more comprehensive metabolic assessment and discover potential markers for the diagnosis of gout. Our metabolomics study may provide a better understanding of the gout pathological mechanism, which may support the clinical diagnosis of gout.

## 2. Experimental and Methods

### 2.1. Patients

A total of 35 patients (45.3±1.8 years) diagnosed with gout according to the latest clinical criteria for the classification of gout proposed by the ACR and EULAR in 2015 were enrolled in this study at the Clinic of Zhejiang Chinese Medical University. The 29 age- and gender-matched healthy Chinese volunteers (43.1±1.6 years) were selected as controls. The age distribution plots of patients and controls were created in supplementary material (Figure S1). Body mass index, blood urate, blood urea nitrogen, erythrocyte sedimentation rate, and serum creatinine were assessed for each arthritic patient (Table 1). Subjects with liver or renal pathology, gastrointestinal disease, endocrine disorders, diabetes mellitus, and drug abuse were excluded. The trial was approved by the ethics committee of Zhejiang Chinese Medical University, and every subject has given informed consent.

### 2.2. Instruments and Reagents

An Agilent 7890/5975C gas chromatograph–mass spectrometer (Agilent Technologies, Santa Clara, CA, USA) was used for acquisition of metabolic data. A Labconco CentriVap system (Labconco, Kansas City, MO, USA) was used to concentrate and dry the metabolite extracts. Ultrapure water was provided by a Milli-Q system (Millipore Corp, Millipore, MA, USA).

Acetonitrile used as the extraction solvent was from Merck (Fairfield, OH, USA). Methoxyamine, methyl-trimethylsilyl-trifluoroacetamide (MSTFA), urease, and pyridine were provided by Sigma-Aldrich (St. Louis, MO, USA). Standards for metabolite identification were purchased from Sigma-Aldrich (St. Louis, MO, USA).

### 2.3. Collection and Storage of Samples

Morning and midstream urine were collected from all the participants. After settling for approximately 10 min, the supernatant was placed in a 1.5 ml Eppendorf vial and then stored at -80°C until use. There were no dietary or other restrictions for the sample collection [20].

### 2.4. Sample Preparation

After thawing, 50 *μ*L of the urine sample was mixed with 80 *μ*L of urease solution (10 mg/mL). The solution was hydrolyzed in a 37°C water bath for 15 min. As the mixture cooled, 500 *μ*L of cold acetonitrile (-20°C) was added. Subsequently, every mixture was vortexed thoroughly for 30 s, settled for 10 min, and centrifuged for 10 min to precipitate the protein (12000g, 4°C). Next, 500 *μ*L of the supernatant was withdrawn and lyophilized under vacuum using the Labconco CentriVap system. Before analysis, the lyophilized metabolites were dissolved in 50 *μ*L of methoxyamine pyridine solution (20 mg/mL) and then reacted for 90 min in a 37°C water bath. As the solution cooled, 50 *μ*L of MSTFA was added for trimethylsilylation for 60 min. After derivatization, the supernatant was transferred to a vial insert for analysis. To test the repeatability and reliability of the data, preparation of quality control (QC) samples and randomization of the study samples are crucial [21]. Equal parts of each urine sample were mixed together to prepare QC samples and then were processed together with study samples by using the same pretreatment method.

### 2.5. GC-MS Analysis

GC–MS analysis was performed on an Agilent 7890/5975C GC–MS system with a 30 m *∗* 0.25 mm *∗* 0.25 *μ*m DB5-MS fused silica capillary column (J&W Scientific, Folsom, CA, USA). High-purity helium (99.9996%) was used as the carrier gas at a constant flow rate of 1.2 mL/min. The sample injection volume was 1 *μ*L, and the split ratio was 10 : 1. The injection temperature was 300°C, and the transfer line temperature was maintained at 280°C. The column temperature was initially set at 70°C for 3 min and then increased to 300°C at 5°C/min and held for 5 min. The mass scan was set from 33 to 600 with a scan speed of 2 scans/s. The solvent cut time was 4.8 min. The analysis order of the gout and volunteer samples was random, and the QC sample was injected every 7 samples within a batch.

### 2.6. Data Processing and Statistical Analysis

GC-MS data were exported in the NetCDF format using the format conversion of the Agilent GC/MSD ChemStation Software (Agilent Technologies, Santa Clara, CA, USA). Then, QC data were imported into AMDIS 2.62 (NIST, Boulder, CO, USA) for peak identification and repetitive peak analysis to obtain a compound table with retention time and quantitative ion of each peak. As the compound table was established, the GC/MSD ChemStation was employed for peak integration of all samples. A data matrix was formed which was composed of the retention times, m/z values, and corresponding peak areas for subsequent statistical analysis. Total peak area normalization was performed on the data to remove systematic variations. The relative intensity of each peak was determined by dividing the peak intensity to the total intensity of the corresponding sample.

The resultant three-dimensional matrix encompassing the peak indices (retention time-m/z pairs), sample names (observations), and normalized peak areas (variables) was imported into SIMCA-P V11.0 software (Umetrics AB, Umea, Sweden) for multivariate statistical analysis to observe the metabolic variations between samples. The orthogonal partial least squares discriminant analysis (OPLS-DA) was applied to classify two groups of samples based on the metabolic data. The significantly altered metabolites between the urine samples from gout patients and healthy controls were screened out according to the variable importance for project (VIP > 1) values and Student's t-test (SPSS 18.0, International Business Machines Corp., Armonk, NY, USA) with statistical significance (p<0.05). The differential metabolites were subjected to binary logistic regression analysis (SPSS18.0) to establish a diagnostic model for gout, and the diagnostic capacity was evaluated by drawing receiver operating characteristic.

### 2.7. Identification of Metabolites

The candidate metabolites were first processed in the AMDIS software for deconvolution to exclude the interference of overlapping peaks. Next, the pure mass spectra were performed in the NIST05 software (National Institute of Standards and Technology, Gaithersburg, MD, USA) for similarity searches by comparing the mass fragments with those present in commercially available databases. The metabolites with similarity greater than 80% were regarded as structurally identified using mass spectral library search, and some of these metabolites were verified using commercial standards.

## 3. Results

### 3.1. Metabolic Profiling of Urine Samples

**Figure 1:**
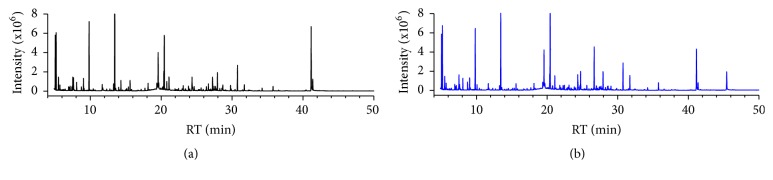
The representative urinary metabolic profiles of healthy control (a) and gout patient (b).

The high abundance of urea in urine samples is unfavorable for GC-MS analysis; thus, urea was hydrolyzed by urease before analysis [22]. In addition, most of the metabolites in urine sample are nonvolatile which precludes direct analysis by GC-MS; thus urine sample has to be derivatized to increase the volatility of metabolites. Silylation is a commonly used derivatization method that is perfectly fitted for untargeted approaches [23]. In this study, a two-step derivatization protocol involving oximation followed by trimethylsilylation was used, which can modify the structures of a range of metabolites with diverse functional groups (hydroxyl, carboxylic acid, amine, amide, imine, thiol, and phosphate groups). Representative GC-MS TICs of urine samples from healthy controls and patients with gout are shown in Figure 1.

### 3.2. Analytical Characteristics of the Urine Metabolic Profiling Method

**Figure 2:**
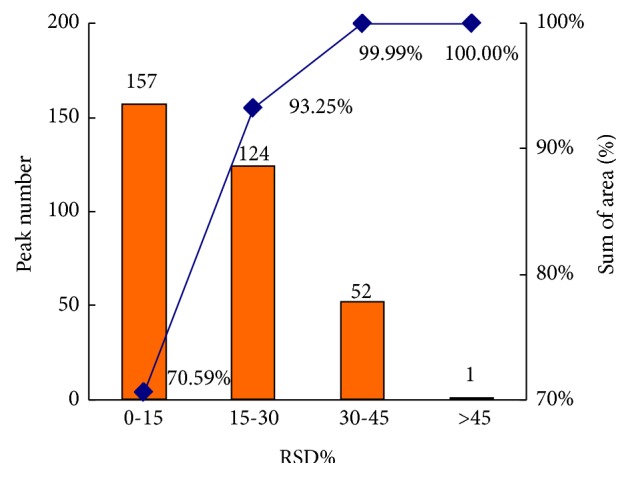
Repeatability of the metabolomics study using GC-MS. RSD distribution plot of all metabolites in QCs was calculated. The column represents the peak number within the specific RSD range, and the line represents the percentage of cumulative peak area within the specific RSD range, respectively.

After peak integration, 334 peaks were detected in the urine samples. To monitor the stability and reproducibility of the analytical conditions, one QC sample was injected every seven samples, and a total of nine QC samples were analyzed throughout the analytical batch. The relative standard deviation (RSD) is calculated by dividing the standard deviation by the average. It reflects the dispersion degree of a set of data and is always used to measure the precision of an analytical procedure. A higher RSD represents a higher variability. The RSD of each peak in nine QC samples was determined to evaluate the reproducibility of the analytical method, and 84.1% of the peaks had RSDs less than 30% and accounted for more than 93.2% of the total peak area (Figure 2), which demonstrated a good reproducibility of the method. Further, OPLS-DA was performed to explore the relations between all samples based on the metabolic data and clarify the metabolic changes in the urine of patients.

### 3.3. Screening of Important Metabolites

**Figure 3:**
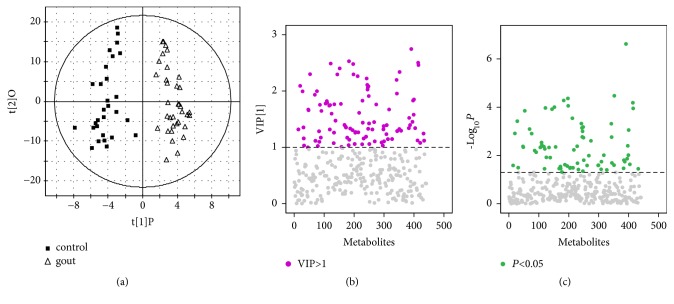
(a) OPLS-DA score plot of gout patients and healthy controls based on the urinary metabolic profiles, (b) VIP of OPLS-DA, and (c)* P*-value of t-test of urinary metabolite in gout patients and healthy controls.

To discover differentially expressed metabolites in gout patients, the metabolic data were processed using OPLS-DA to remove the information unrelated to classification. The cumulative values of R2Y (R2Y=0.911) and Q2Y (Q2Y=0.755) indicated that the model has a good predictive and interpretative capacity. In the OPLS-DA score plot, patients were obviously distinguished from healthy controls (Figure 3(a)). Metabolites at both ends of the V-plot were considered important for classification (Figure 3(b)).

76 variables were considered to be associated with gout with VIP > 1 and p<0.05 between the gout patients and the controls. The NIST05 software was used to identify the possible structures of the metabolites, and the reliability of the results was verified by standard products. Finally, 30 metabolites were identified (Table 2), including ethanolamine, phenylethanolamine, glycolate, glycerol, galacturonic acid, stearate, succinate, fumarate, isoleucine, serine, glycine, threonine, aspartate, pyroglutamate, phenylalanine, propylene glycol, 5-hydroxy-l-tryptophan, l-tryptophan, 5-hydroxyindole-3-acetate, beta-lactate, threonate, d-lyxose, ribitol, sorbitol, d-allose, gluconate, uracil, urate, creatinine, and isoxanthopterin.

### 3.4. Discovering Potential Markers for Diagnosis of Gout

**Figure 4:**
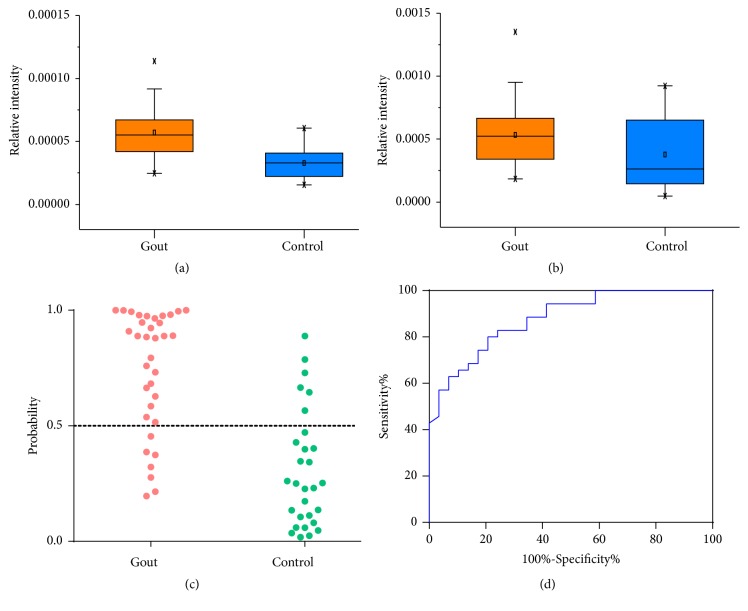
(a) The levels of isoxanthopterin in the urine samples from gout patients and controls, (b) the levels of urate in the urine samples from gout patients and controls, (c) probability of gout patients and controls based on the combination of urine urate and isoxanthopterin, and (d) ROC analysis for the diagnosis of gout using the combination of urate and isoxanthopterin (AUC=0.879).

The differential metabolites between patients and controls were regarded as potential markers, and the binary logistic regression was performed to establish a diagnostic model for gout. Based on the step forward method (likelihood ratio), a model including two metabolites was constructed. Urate and isoxanthopterin, which were detected at increased levels in the urine of gout patients, were enrolled in the model (Figures 4(a)-4(b)). The combination of urate and isoxanthopterin performed high classification accuracy for gout patients and healthy controls, which were 80.0% and 79.3%, respectively (Figure 4(c)). ROC curve showed the combination of urate and isoxanthopterin can effectively discriminate two groups of samples with the AUC of 0.879 (Figure 4(d)).

## 4. Discussion

**Figure 5:**
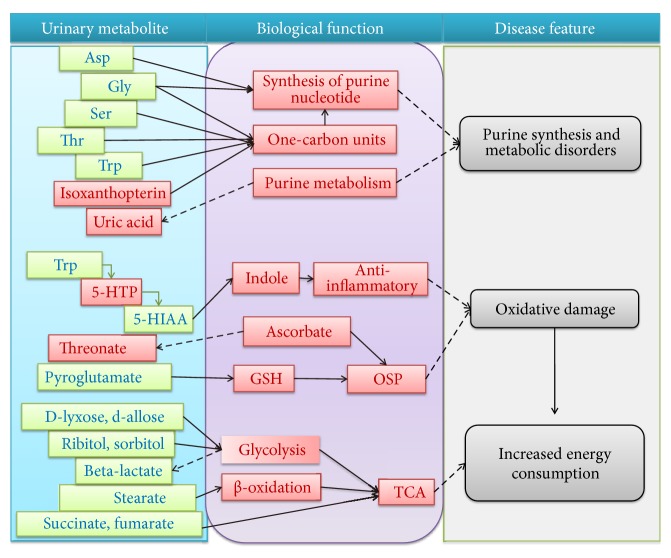
Significantly changed urinary metabolites of gout patients and the biological functions. The metabolites marked in red indicate significantly increase in gout patient, and blue indicates decrease. Asp, aspartate; Gly, glycine; Ser, serine; Thr, threonine; Try, l-tryptophan; 5-HPT, 5-hydroxy-l-tryptophan; 5-HIAA, 5-hydroxyindole-3-acetate; GSH, glutathione; OSP, antioxidation; TCA, tricarboxylic acid cycle.

In this study, we were able to discriminate gout patients from healthy controls in an OPLS-DA model based on urinary metabolites, and 30 significantly different metabolites were identified which were mainly involved in purine nucleotide synthesis, amino acid metabolism, purine metabolism, lipid metabolism, carbohydrate metabolism, and tricarboxylic acid (TCA) cycle (Figure 5).

We found that the level of urine urate was higher in patients with gout than in controls. During attacks of acute gout, the levels of glucocorticoids and inflammatory factors increase owing to the state of organism stress, which may promote the urinary excretion of urate and reduce the level of blood urate [24, 25]. Detection of urine urate levels may have a great meaning in clinical applications for the assessment of the state of gout disease. Slight change in creatinine level was likely due to the fluctuations in kidney function caused by tubular defects and the excretion of urate in gout patients [26].

Urate is produced by the metabolism of purine nucleotides. Urate levels can be affected by abnormal nucleotidase generation and catabolism in the human body [27, 28]. Purine nucleotides are synthesized mainly through a de novo synthesis pathway in which amino acids and energy are consumed. Glycine, aspartate, and one-carbon unit are the raw materials for purine nucleotide synthesis, and glycine, serine, threonine, and tryptophan catabolism is the main source of the single carbon unit. Isoxanthopterin is a degradation product of folic acid, and the reductive product of folic acid is the carrier of one-carbon units. Our study suggested that reduced amino acid levels and increased isoxanthopterin level in gout patients may indicate a disorder of the metabolism of one-carbon units and nucleic acid synthesis and appears to be associated with the acceleration of purine nucleotide synthesis in patients.

Inflammatory factors produced during the development of gout may stimulate the requirement of energy [29]. Body's energy is mostly obtained from the TCA cycle [30], and it was indicated that several intermediate metabolites in the TCA cycle, such as succinate and fumarate, were significantly reduced in the urine samples of gout patients, for they were excessively consumed to produce energy. Fatty acid can generate energy through *β*-oxidation, and it was observed that the level of stearate was also downregulated in gout patients. In addition, branched-chain amino acids, another source of energy, were also decreased to keep up with body's energy demands. Our study suggested that the levels of branched-chain amino acids, fatty acids, and intermediate metabolites in the TCA cycle that directly or indirectly produce energy were all downregulated to meet the growing demand for energy in gout patients.

Pyroglutamate can be converted into glutathione (GSH) which is a primary antioxidant. Pyroglutamate was detected at significantly decreased levels in patients with gout. It has been reported that the oxidative of urate could promote the consumption of GSH [31]. The excessive consumption of GSH may lead to a lower level of pyroglutamate in the urine of gout patients.

Tryptophan is an aromatic amino acid and has a role in humoral immunity [32]. Tryptophan and its intermediate metabolites participate in the immune response of the body by means of affecting the function of the T cell [33]. In this study, tryptophan and its downstream products 5-hydroxyindole-3-acetate were both downregulated in the urine of patient which may be associated with the abnormality of immunological response of patients.

Intestinal flora, as significant regulators, participates in the human body's metabolic processes. Owing to the lack of metabolic enzymes for benzene compounds in organisms, phenyl-containing compounds are degraded mainly by intestinal flora [34]. In our study, the level of phenylalanine was decreased in the urine of gout patients, which were likely related to a disorder of gut flora. Therefore, an imbalance in intestinal flora is likely to occur during the development of gout.

High urate level is a predominant feature of gout patients. the urate level is considered as an important reference indicator for the diagnosis of gout clinically, but the diagnostic accuracy is poor based on the urate level alone. Most of the hyperuricaemia population would not develop gout, and occasionally gout can attack patients with normal urate level. The urate crystal examination is a gold standard for gout diagnosis, but the examination is an invasive examination and requires strong operation skill of doctor, so the clinical applications have been limited. Therefore, it is necessary to find a more simple, noninvasive, and accurate diagnosis index of gout. In the study, some significantly changed metabolites in the urine of gout patients were discovered and these metabolites may have potentials in the diagnosis of gout. With the binary logistic regression analysis, a model based on the levels of urate and isoxanthopterin was constructed to distinguish gout patients from controls, and the model can effectively discriminate between two groups. ROC curve analysis showed the combination of urate and isoxanthopterin provided good performance for the diagnosis of gout.

## 5. Conclusion

Obvious metabolic differentiation between gout patients and healthy controls was observed by using GC-MS-based urinary metabolomics study. The altered metabolites in the urine of gout patients mainly include amino acids, carbohydrates, organic acids, and their derivatives. These metabolites were primarily associated with perturbations in purine nucleotide synthesis, amino acid metabolism, purine metabolism, lipid metabolism, carbohydrate metabolism, and TCA cycle. Binary logistic regression and ROC curve analysis showed the combination of urate and isoxanthopterin can effectively discriminate the gout patients from controls. Thus, the urinary metabolomics study is an efficient tool for a better understanding of the metabolic changes and discovery of metabolite markers for gout, which may support the clinical diagnosis and management of gout. However, there are also potential limitations of our study. Some bias may exist due to the relatively small population included in our study and large sample validation is still required in the future works.

## Figures and Tables

**Table 1:** Demographic and clinical chemistry characteristics of gout patients.

	Group		*P* value
	Gout patients (N=35)	Healthy controls (N=29)	
Age (years)	45.3±1.8	43.1±1.6	*P*>0.05
Male/female ratio	34/1	27/2	*P*>0.05
Body mass index (kg/m2)	24.4±0.3	23.7±0.4	*P*>0.05
Blood urate (*μ*mol/L)	434.8±14.8	-	-
Blood urea nitrogen (mmol/L)	6.5±0.3	-	-
Serum creatinine (*μ*mol/L)	76.8±3.6	-	-
ESR (mm/h)	^#^13.4±1.7	-	-

Note: data are presented as mean ± SEM; ESR means erythrocyte sedimentation rate; # represents the information on ESR was available for 33 patients.

**Table 2:** Significantly changed urinary metabolites in patients with gout.

Metabolite	^*a*^VIP	^b^P	Trend	Metabolic pathway
Ethanolamine	1.82	2.91E-03	↓	Glycerophospholipid metabolism
Phenylethanolamine	1.67	9.76E-03	↓	Glycerophospholipid metabolism
Glycolate	2.3	1.41E-04	↓	Glycerophospholipid metabolism
Glycerol	1.74	4.35E-03	↓	Glycerolipid metabolism
Galacturonate	1.32	2.52E-02	↑	Unknowm
Stearate	1.34	2.34E-02	↓	Unsaturated fattyacids metabolism
Succinate	1.6	1.20E-02	↓	TCA cycle
Fumarate	1.47	1.23E-02	↓	TCA cycle
Isoleucine	1.75	6.29E-03	↓	Valine, leucine and isoleucine metabolism
Serine	1.78	4.63E-03	↓	Glycine, serine and threonine metabolism
Glycine	2.49	1.08E-04	↓	Glycine, serine and threonine metabolism
Threonine	1.32	3.57E-02	↓	Glycine, serine and threonine metabolism
Aspartate	2.53	5.26E-05	↓	Alanine,aspartate and glutamate metabolism
Pyroglutamate	1.31	0.027421134	↓	Alanine, aspartate and glutamate metabolism
Phenylalanine	1.44	2.10E-02	↓	Phenylalanine metabolism
Propylene glycol	2.09	1.22E-03	↓	Propanoate metabolism
5-Hydroxy-l-tryptophan	1.39	1.81E-02	↑	Tryptophan metabolism
L-Tryptophan	1.45	1.37E-02	↓	Tryptophan metabolism
5-Hydroxyindole-3-acetate	1.58	9.73E-03	↓	Tryptophan metabolism
Beta-lactate	2	1.24E-03	↓	Glycolysis metabolism
Threonate	1.26	3.35E-02	↑	Ascorbate and aldarate metabolism
D-lyxose	1.33	4.48E-02	↓	Pentose and glucuronate interconversions
Ribitol	1.76	2.48E-03	↓	Pentose and glucuronate interconversions
Sorbitol	1.95	1.74E-03	↓	Fructose and mannose metabolism
D-allose	1.34	3.42E-02	↓	Fructose and mannose metabolism
Gluconate	2.34	3.37E-05	↑	Pentose phosphate pathway
Uracil	1.76	4.67E-03	↓	Pyrimidine metabolism
Urate	1.39	1.81E-02	↑	Nucleotide metabolism
Creatinine	1.61	5.89E-03	↓	Arginine and proline metabolism
Isoxanthopterin	2.75	2.40E-07	↑	Unknown

“^*a*^VIP” value was obtained from OPLS-DA with a threshold of 1.0; “^*b*^P”values were calculated from Student *t-*test; “↓” means the level was decreased in the urine of gout patients, and “↑” means the level was increased in gout patients; “Unknown” means the metabolic pathway of corresponding metabolite was nonexistent in the KEGG database.

## Data Availability

The data used to support the findings of this study are available from the corresponding author upon request.

## Supplementary Materials

Supplementary Materials Figure S1: the age distribution plot for gout patients and controls. Figure S2: the levels of important metabolites in the urine samples from gout patients and controls. Table S1: significantly changed urinary metabolites in patients with gout.
