# Supplementary material for: Urinary Metabolomics Study of Patients with Gout Using Gas Chromatography-Mass Spectrometry
Source: Biomed Res Int. 2018 Oct 16;2018:3461572. doi: 10.1155/2018/3461572 (PMC6206583; doi:10.1155/2018/3461572)
Supplement: Supplementary Materials — Figure S1: the age distribution plot for gout patients and controls. Figure S2: the levels of important metabolites in the urine samples from gout patients and controls. Table S1: significantly changed urinary metabolites in patients with gout. [file 3461572.f1.pdf]

Urinary metabolomics study of patients with gouty arthritis using gas chromatography-mass spectrometry

Qianqian Li[a,1], Shuangshuang Wei[a,1], Dehong Wu[b], Chengping Wen[a,*], Jia Zhou[a,*]

[a] *College of Basic Medical, Zhejiang Chinese Medical University, 548 Binwen Road, Hangzhou 310053, China*

[b] *The Second Affiliated Hospital of Zhejiang Chinese Medical University, 318 Chaowang Road, , Hangzhou 310053, China*

[1]These authors contributed equally to this work

*Corresponding authors:

Jia Zhou, Phone: 86-571-86613587; Fax: 86-571-86613587; E-mail: zhoujia@zcmu.edu.cn;

Chengping Wen, Phone: 86-571-86613131; Fax: 86-571-86613131: E-mail: wengcp@163.com

Supplementary materials

Table S1 Significantly changed urinary metabolites in patients with gout

Metabolite	Retention Time (min)	PubChem ID	Identification Level [a]
Ethanolamine	13.28	700	1
Phenylethanolamine	27.2229	1000	2
Glycolate	8.0842	5460308	1
Glycerol	13.595	753	1
Glycerate	15.0457	439194	1
Stearate	34.2319	5281	1
Succinate	14.6031	1110	1
Fumarate	15.5835	444972	1
Isoleucine	10.8297	6306	1
Serine	13.0235	5951	1
Glycine	14.3231	750	1
Threonine	14.0147	6288	1
Aspartate	18.537	5960	2
Pyroglutamate	19.6691	7405	1
Phenylalanine	22.115	6140	1
Propylene glycol	6.0702	1030	2
5-hydroxy-l-tryptophan	26.9568	144	2
L-Tryptophan	33.3073	6305	1

5-Hydroxyindole-3-acetate	33.6341	1826	2
Beta-lactate	9.9734	91435	2
Threonate	20.3778	151152	2
D-lyxose	23.0948	439240	1
Ribitol	24.4425	6912	1
Sorbitol	28.6909	5780	1
D-allose	32.2984	12285879	2
Gluconate	29.2571	10690	2
Uracil	15.1452	1174	1
Urate	32.5264	1175	1
Creatinine	21.2068	588	2
Isoxanthopterin	33.0729	10729	2

[a] Identification level for metabolites according to Metabolomics Standards Initiative (MSI). 1: validation using reference standards; 2: identification using mass spectral library search.

Supplementary materials

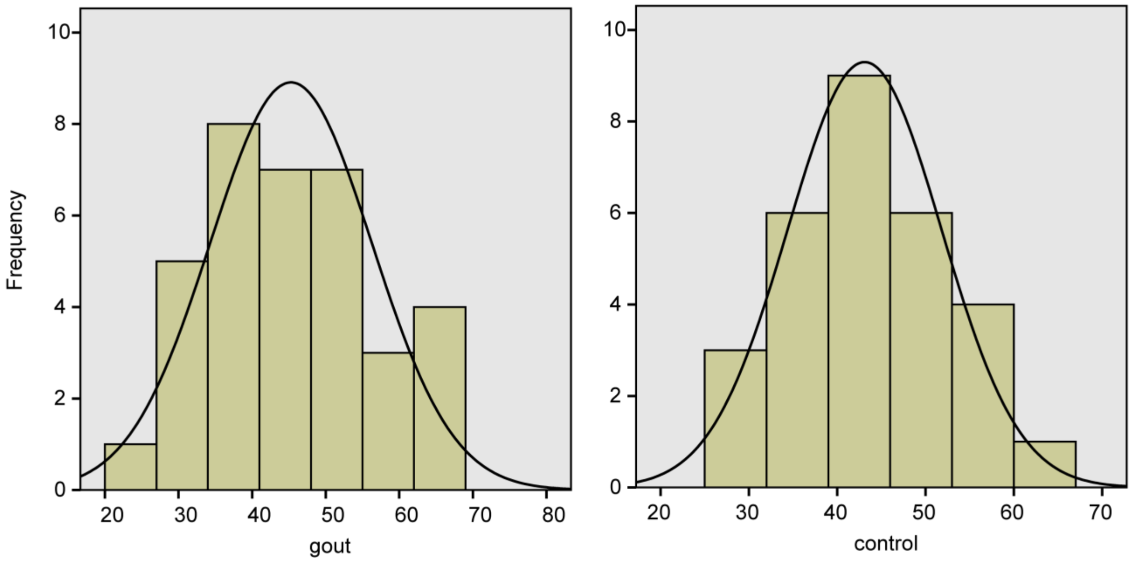

Fig. S1. The age distribution plot for gout patients and controls

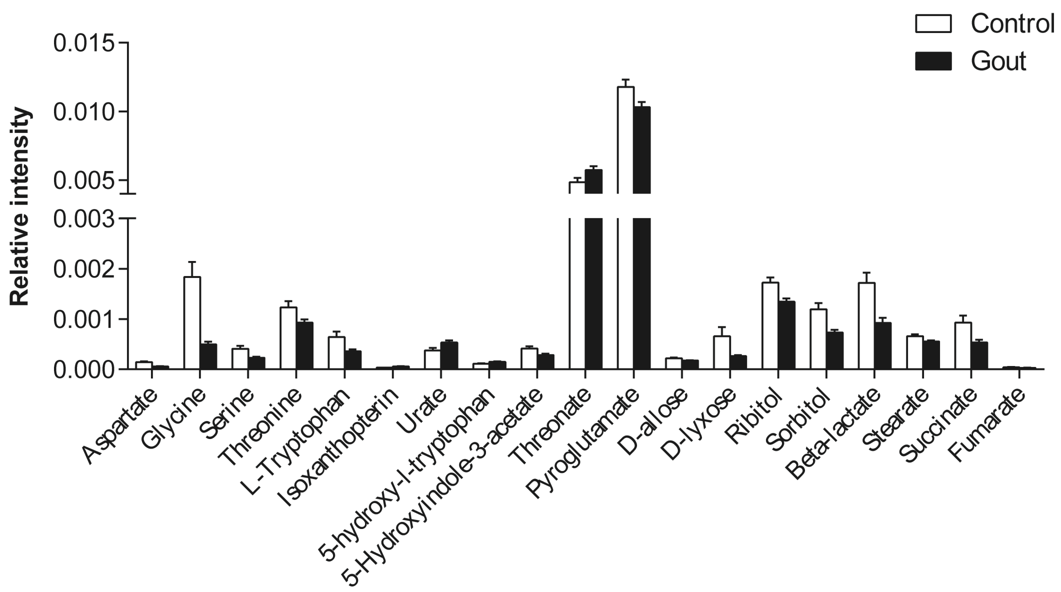

Fig. S2 The levels of important metabolites in the urine samples from gout patients and controls
